# Influence of Cocoa Liquor Origin on Cocoa Butter Composition and Color, Texture, and Rheological Properties of Milk and Dark Chocolates: A Preliminary Study

**DOI:** 10.1002/fsn3.72130

**Published:** 2026-07-29

**Authors:** Onur Ozdemir, Hasene Keskin‐Cavdar, Suleyman Polat, Mustafa Umit Unal

**Affiliations:** ^1^ Department of Food Engineering, Faculty of Engineering Cukurova University Adana Turkiye; ^2^ Department of Food Engineering, Faculty of Engineering Gaziantep University Gaziantep Turkiye

**Keywords:** bending force color analysis, chocolate manufacturing, fatty acids, shear stress, viscosity

## Abstract

This study presents a preliminary evaluation of the physicochemical characteristics of cocoa liquors from Ecuador, Ghana, and Côte d'Ivoire, and assesses their impact on the color, texture, and rheological properties of milk and dark chocolates. The moisture content, cocoa butter content, and color properties of cocoa liquors from three geographical locations were analyzed. The cocoa butters extracted from cocoa liquors were examined for solid fat content (SFC), fatty acid composition, free fatty acidity, and peroxide value. Milk and dark chocolates were prepared differing only in the origin of the cocoa liquor and tested for color, texture, and rheological properties. Cocoa liquors differed in moisture, fat content, and color depending on their geographical origin (*p* < 0.05). Stearic (C18:0), oleic (C18:1), and palmitic (C16:0) acids were the most prevalent fatty acids in all cocoa butters, constituting more than 90% of total fatty acids. SFC of the cocoa butters showed no significant variation. The application of cocoa liquors from different origins resulted in comparable color, texture, or rheological properties in either milk or dark chocolates. However, differences were observed between milk and dark chocolates, with dark chocolates exhibiting lower luminosity and chroma and greater penetration hardness. All samples exhibited a time‐dependent decrease in viscosity, indicating that the cocoa liquor source did not markedly affect the flow characteristics of chocolate. PCA and HCA clearly differentiated cocoa liquors according to geographical origin, whereas chocolate samples were primarily grouped by formulation type (milk or dark chocolate). In conclusion, this preliminary study suggests that although geographical origin influences the physicochemical characteristics of cocoa liquors, its effect on the textural and rheological properties of milk and dark chocolates appears to be limited. These findings are based on a single production batch and require confirmation through independent process replicates.

## Graphical abstract

This study compared cocoa liquors from Ecuador, Ghana, and Côte d'Ivoire and evaluated their effects on the color, textural, and rheological properties of milk and dark chocolates. The results showed that cocoa origin influenced cocoa liquor characteristics and contributed to differences in chocolate quality.

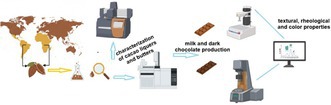

## 1 Introduction

Cocoa ( *Theobroma cacao* L.) is a significant agricultural product that originated in Central and South America and is now cultivated globally since it is the only source of cocoa butter and powder for the $200 billion worldwide confectionery market. Cocoa ( *Theobroma cacao* L.) is a major tropical crop originating from Central and South America and widely cultivated in tropical regions worldwide. It is the primary commercial source of cocoa butter and cocoa powder, which are essential ingredients in the global confectionery industry, a market valued at over 180 billion USD and continuing to grow in recent years (ICCO 2024; Grand View Research 2023). Cocoa beans are the primary raw material used in chocolate production and impact the sensory, physical, and rheological properties of chocolate products (Sari et al. 2023). Cocoa liquor is a critical intermediate product that is produced by roasting and grinding fermented and dried cocoa beans and consists of fundamentally cocoa butter and cocoa solids, in characteristic proportions. The physicochemical and aromatic characteristics of cocoa liquor directly influence the color, flavor, texture, and rheological properties of chocolate. Cocoa bean origin influences these properties due to its close association with genetic characteristics and environmental growing conditions (Liu et al. 2017). The differences in cocoa properties can be linked to the inherent genetic composition of the bean, botanical origin, location of growth, and growing conditions, such as climate, sunshine and rainfall, soil conditions, ripening, harvesting, and bean fermentation, which all affect the final characteristics (Kongor et al. 2016).

Chocolate is a complex multiphase suspension in which cocoa solids and sugar particles are spread throughout a continuous cocoa butter phase (Fernandes et al. 2013). Chocolate is generally classified as dark, milk, or white, depending on changes in the composition of cocoa, fat, and milk components (Zarić et al. 2024). The general impression of chocolate's quality is primarily linked to its taste and texture, especially its ability to melt quickly in the mouth while maintaining its firmness at room temperature, leaving a smooth and homogeneous sensation (Afoakwa et al. 2007). Chocolate production involves a series of sequential process steps, including mixing, particle size reduction, conching, standardization, and tempering. These processes critically influence particle size distribution, fat‐solid interactions, viscosity, and the final textural properties of the chocolate (Afoakwa 2010; Beckett et al. 2017). While blending and refining processes are used to ensure cocoa butter is effectively coated with solid particles, the conching process is considered a crucial step in reducing moisture, removing unwanted volatile compounds, and enhancing flavor development.

The increasing global demand for chocolate has intensified research interest in cocoa‐based products and their functional, compositional, and processing‐related properties. Cocoa liquor is one of the primary raw materials used in the manufacture of milk and dark chocolate and exerts a decisive influence on color, texture, and rheological properties. Previous studies have demonstrated that cocoa origin may influence the compositional and quality characteristics of cocoa products, including phenolic compounds, methylxanthines, organic acids, and mineral composition (Araujo et al. 2014). Such studies mainly focus on the compositional and nutritional characterization of cocoa beans and liquors. In contrast, the present study emphasizes the technological and macro‐physical properties of cocoa liquors and chocolates, particularly those related to industrial processing behavior and product quality. Therefore, the present study aimed to investigate cocoa liquors from three origins (Ghana, Côte d'Ivoire, and Ecuador) with respect to moisture content, fat content, color parameters, free fatty acid content, peroxide value, and fatty acid composition. The Ghana, Ivory Coast, and Ecuador liquors were selected to represent major global cocoa supply origins and distinct cocoa types used in the chocolate industry. Ghana and Ivory Coast represent bulk cocoa‐producing regions that dominate global supply, while Ecuador represents a fine‐flavor cocoa origin characterized by different genetic and compositional attributes. The study focuses on the comparative physicochemical evaluation of these cocoa liquors; sensory and aroma analyses were not included. In addition, milk and dark chocolates produced from these cocoa liquors were analyzed for color, texture, and rheological properties to assess the effect of cocoa liquor origin on chocolate quality attributes.

## 2 Materials and Methods

Cocoa liquors used in this study, supplied by Rotel Co. (Turkey), were obtained from Ghana, Ivory Coast, and Ecuador as representative origins of major cocoa supply systems. Ghana and Ivory Coast were used as bulk cocoa references, while Ecuador was included as a fine‐flavor cocoa origin. Powdered sugar was purchased from a local market. Cocoa butter and cocoa powders were purchased from Altınmarka (Turkiye). Milk powder and demineralized whey powder were obtained from Enka Milk (Turkiye). Sunflower lecithin was supplied by Yılmaz Kimya (Turkiye), and vanillin was obtained from Univar (USA). Based on these cocoa liquors, six chocolate samples (three milk chocolates and three dark chocolates) were produced. Within each chocolate category, the formulations remained constant, with the only variable being the geographical origin of the cocoa liquor. Hexane, phenolphthalein, potassium hydroxide (KOH), chloroform, potassium iodide (KI) used in chemical analyses were purchased from Merck (Germany), while ethanol, diethyl ether obtained from Isolab (Germany).

### 2.1 Characterization of Cocoa Liquors

Cocoa liquors sourced from Ghana and Ivory Coast are generally classified as bulk cocoa and are predominantly associated with Forastero‐type cocoa varieties cultivated in West Africa. Ecuador was included as a representative fine‐flavor cocoa origin. The study was designed to compare the physical and technological properties of cocoa liquors and chocolates produced from these geographical origins. The scope of this study was limited to physical and technological characterization; therefore, bioactive compound and detailed compositional analyses were not included.

#### 2.1.1 Moisture Analysis

Moisture content was determined according to AOAC method 931.04 (AOAC 2020). 5 g of the sample was placed in a petri dish and spread. Then the petri dishes were dried in an oven at 105°C until they reached a constant weight. For the calculation, Equation (1) was used.

(1) $$\begin{array}{c} \text{moisture content}\;\left(\%,\frac{g}{g}\right)=\\ \frac{\text{weight of sample before drying}-\text{weight of sample after drying}\;}{\text{weight of the sample before drying}}\\ \times 100 \end{array}$$

#### 2.1.2 Cocoa Butter Content

20 g of cocoa liquor was mixed with 200 mL of hexane in an Erlenmeyer flask and shaken in a shaking incubator (New Brunswick Innova 40 Series, Germany) for 6 h at 55°C. The mixture was then placed in Falcon tubes and centrifuged at 25°C for 20 min at 6000 rpm (Eppendorf, Centrifuge 5810R, VWR International LLC, Radnor, PA, USA) to collect supernatant. A rotary vacuum evaporator (Hei‐VAP Advantage HL/G1; Heidolph Instrument GmbH & Co. KG) was used to remove hexane at 40°C (Sirisompong et al. 2011).

(2) $$\text{Cocoa butter content}\;\left(\%,\frac{g}{g}\right)=\frac{\text{weight of extracted oil}\;\left(g\right)}{\text{weight of cocoa liquer}\;\left(g\right)}\times 100$$

#### 2.1.3 Color Analysis

Color parameters were analyzed via a HunterLab ColorFlex EZ spectrophotometer (USA) according to the CIELAB system, and L*, a*, and b* values were measured. Hue angle (*h*°) and chroma (C*) were calculated as follows (Briones and Aguilera 2005):

(3) $$h^{{}^{\circ}}=\arctan\left(\frac{b^{*}}{a^{*}}\right)$$

(4) $$C^{*}={\left[{\left(a^{*}\right)}^{2}+{\left(b^{*}\right)}^{2}\right]}^{0.5}$$

### 2.2 Characterization of Cocoa Butters

#### 2.2.1 Solid Fat Content (SFC)

The solid fat content of extracted cocoa butter was determined by differential scanning calorimetry (DSC; Perkin Elmer DSC‐6, USA). DSC was calibrated using indium (melting point 156.6°C, enthalpy of fusion 28.45 J g^−1^). The purge gas utilized was nitrogen at 40 mL/min. Samples (5–10 mg) were completely melted at 80°C, sealed in aluminum pans, and analyzed against an empty sealed pan. The thermal history of the samples was eliminated by holding at 80°C for 10 min. The temperature was then decreased to −60°C at 5°C/min and held for 10 min. After that, the sample was reheated to 80°C at 5°C/min. SFC values at different temperatures were calculated from DSC heating thermograms (Tieko and Aparecida 1995).

#### 2.2.2 Fatty Acid Composition

A Shimadzu GC17A gas chromatograph (Japan) with a flame ionization detector and BPX capillary column (30 m × 0.22 mm i.d. × 0.25 μm film) was used to examine the fatty acid methyl esters from fatty acids. The injector and detector temperatures were set to 225°C and 250°C, respectively. The oven protocol consisted of: 60°C for 1 min, followed by a ramp to 170°C at 10°C/min, then to 230°C at 3°C/min, and maintained for 15 min. Nitrogen served as the carrier gas at a flow rate of 1 mL/min. FAMEs were identified by comparing retention times against standards (Keskin Çavdar et al. 2023).

#### 2.2.3 Free Fatty Acidity (FFA)

The AOAC official method 940.28 (2020) was used to measure free fatty acids (FFA) using titration. 5 g cocoa butter dissolved in 50 mL ethanol–diethyl ether (1:1, v/v). The samples were titrated with 0.1 N ethanolic KOH in the presence of phenolphthalein as indicator. FFA was calculated using Equation (5) and expressed in terms of oleic acid.

(5) $$\begin{array}{c} \mathrm{FFA}\%\left(g/g\right)=\\ \frac{\text{used}\;\mathrm{KOH}\;\text{solution}\left(\mathrm{mL}\right)\times \text{normality}\;\mathrm{KOH}\times 0.282}{\text{weight of cocoa butter}\;\left(g\right)}\times 100 \end{array}$$

#### 2.2.4 Peroxide Value (PV)

Peroxide value was determined using the AOAC Standard Method 965.33 (2020) with some modifications. Two grams of melted cocoa butter were dissolved in 10 mL of chloroform, followed by the addition of 15 mL of acetic acid and 1 mL of KI. The flask was capped and kept in the dark for 5 min. Then, 75 mL distilled water and 1 mL starch solution were added. Samples were titrated with 0.01 N sodium thiosulfate until the dark color disappeared. Equation (6) was used to determine the peroxide value:

(6) $$\begin{array}{c} \mathrm{PV}\;\left(\mathrm{meq}\;O_{2}/\mathrm{kg}\;\mathrm{fat}\right)=\\ \frac{\text{volume of sodium thiosulfate used}\;\left(\mathrm{mL}\right)\times \text{Normality of}\;{\mathrm{Na}}_{2}S_{2}O_{3}\times 1000}{\text{weight of the cocoa butter}\;\left(g\right)} \end{array}$$

### 2.3 Chocolate Production and Characterization

#### 2.3.1 Chocolate Production

**FIGURE 1:**
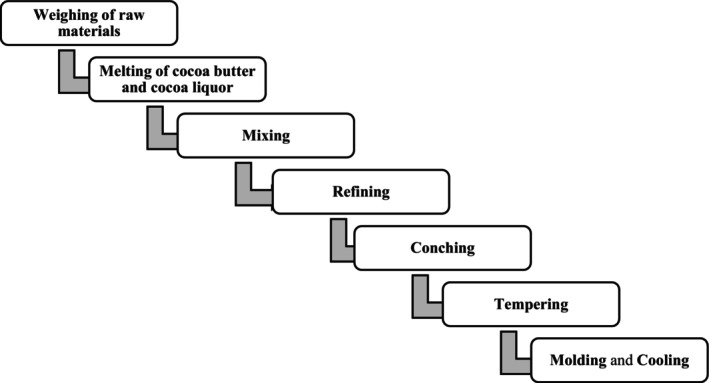
Flow diagram of chocolate production.

Milk and dark chocolates were produced according to the flow diagram shown in Figure 1. All chocolates were prepared in 5 kg batches under identical conditions. Dark chocolate was formulated with cocoa mass (45%), cocoa butter (10%), powdered sugar (44.5%), and sunflower lecithin (0.5%). Milk chocolate contained cocoa mass (12%), cocoa butter (23%), powdered sugar (44.5%), whole milk powder (20%), and sunflower lecithin (0.5%). Cocoa butter and cocoa liquor, being solid at room temperature, were melted prior to weighing. The ingredients were homogenized in an industrial mixer, and subsequently refined to a target particle size of approximately 18 μm using a roller refiner. During refining, roller temperatures were maintained at 35°C–40°C, 30°C–35°C, and 25°C–30°C for the feed, intermediate, and final rollers, respectively, for dark chocolate, while corresponding temperatures for milk chocolate were 28°C–32°C, 25°C–28°C, and 20°C–24°C. Particle size was verified using a micrometer (Mitutoyo Series 293 MDC‐MX Lite, Japan). The refined chocolate mass was conched in three stages using a single‐shaft conche (Bühler Frisse Elkolino). Conching was performed at 60°C for dark chocolate and 50°C for milk chocolate for a total of 4 h. Sunflower lecithin and part of the cocoa butter were added during the first and final stages of conching. Following conching, the chocolate masses were heated to 45°C to eliminate pre‐existing cocoa butter crystal forms and then tempered manually on a tempering workbench maintained at 20°C. The chocolate was spread continuously until its temperature decreased to 26°C–27°C and a suitable molding consistency was achieved. The tempered chocolate was then molded and cooled at 14°C for at least 10 min prior to further analyses.

#### 2.3.2 Physical Characterization of Chocolate Samples

##### 2.3.2.1 Color Analysis

Color of the chocolates was measured using a HunterLab ColorFlex EZ spectrophotometer (USA) as described in Section 2.1.3.

##### 2.3.2.2 Texture Analysis

A TA‐XT Plus texture analyzer (Stable Micro Systems, UK) was employed to assess texture properties of the chocolates. Three‐point bending and penetration tests were applied to milk and dark chocolates prepared with cocoa liquors of Ghanaian, Ecuadorian, and Ivorian origin. The three‐point bending test was performed with the HDP/3PB probe on samples molded into bars (3 mm thickness, 25 mm span). Samples were equilibrated at 25°C for 24 h prior to testing. The test settings were as follows: pre‐test speed of 2 mm/s, test speed of 1 mm/s, post‐test speed of 10 mm/s, and a distance of 15 mm. Hardness was determined with a penetration probe (P/5) and a 5 kg load cell. Two chocolate pieces were stacked for each measurement. The test parameters for hardness were adjusted as follows: 1 mm/s for the pre‐test, 2 mm/s for the test, 10 mm/s for the post‐test, and 5 mm penetration depth. Tests were performed at room temperature (Afoakwa et al. 2008).

##### 2.3.2.3 Rheological Analysis

Rheological properties were measured using a Brookfield DV3T‐RV viscometer (USA) equipped with a TC‐150 water bath (USA). Chocolates were preconditioned in an incubator at 50°C for 1 h 15 min to ensure uniform temperature. Measurements were carried out at 40°C with shear rates ranging from 2 to 48 rpm. Data were recorded at 10 s intervals. Melted samples were transferred to the viscometer's cylindrical chamber, and spindle SC4‐27 was used. Results were recorded automatically (Adewale 2017).

### 2.4 Statistical Analysis

Data obtained from cocoa liquors of three different geographical origins, cocoa butters extracted from these liquors, and chocolates produced with them were subjected to analysis of variance (ANOVA) using SPSS software, version 25 (IBM SPSS Statistics, USA). Tukey's multiple‐comparison test was used to determine significant differences among samples. All measurements were performed in triplicate as technical replicates from a single production batch for each formulation. No independent process replicates were included in the experimental design. Therefore, the present work should be regarded as a preliminary investigation, and the findings should be interpreted accordingly. Principal component analysis (PCA) and hierarchical cluster analysis (HCA) were performed using Origin Lab software version 2019 (Northampton, MA, USA).

## 3 Result and Discussion

### 3.1 Properties of Cocoa Liquors

The results of the moisture content analysis for cocoa liquors originating from three different origins (Ecuador, Ghana, and Côte d'Ivoire) are presented in Table 1. The moisture content of the cocoa liquors ranged from 0.23% to 1.10% (g/g). The highest average moisture content was observed in the Ecuador‐origin liquor with a value of 1.10% (g/g) while the lowest moisture content was found in the Côte d'Ivoire‐origin liquor with a value of 0.23% (g/g) (*p* < 0.05). The moisture content of cocoa liquor is considered to play a critical role in determining product quality and stability, but there is no definitive information on what its optimum range should be. Although industrial practice typically maintains moisture levels between 1.0% and 1.5%, the reported optimal range is 0.8% to 1.8% (Dand 2011). Tuenter et al. (2020) investigated the volatile and non‐volatile profiles of cocoa liquors from West Africa and Ecuador, reporting average moisture contents below 1.5% for both regions. Similarly, Tran et al. (2016) examined the quality characteristics of dark chocolates produced from Vietnamese cocoa liquors with varying acidity levels and found that their moisture contents ranged from 0.4% to 3.9%. In the present study, the measured moisture levels of cocoa liquors originating from Ecuador, Ghana, and Côte d'Ivoire were consistent with previously reported values in the literature, further supporting the notion that moisture contents below approximately 1.5% are typical for liquors of diverse geographical origins.

**TABLE 1:** Moisture content, cocoa butter amount and color properties of cocoa liquors.

Origin	Moisture content (%)	Cocoa butter amount (dry matter basis, %)	L*	*h°*	C*
Ecuador	1.10 ± 0.21^a^	53.14 ± 0.06^b^	14.09 ± 0.03^b^	47.29 ± 0.44^b^	22.25 ± 0.11^b^
Ghana	0.50 ± 0.01^b^	53.81 ± 0.10^a^	14.85 ± 0.02^a^	48.42 ± 0.65^ab^	22.23 ± 0.21^b^
Côte d'Ivoire	0.23 ± 0.03^c^	52.88 ± 0.07^c^	13.36 ± 0.07^c^	49.16 ± 0.83^a^	23.09 ± 0.48^a^

*Note:* Values followed by different letters are significantly different (*p <* 0.05) (Tukey multiple range test). The cocoa liquors represent commercially supplied bulk materials processed under standardized chocolate manufacturing conditions. Values are mean ± standard deviation.

The color parameters of cocoa liquor samples varied significantly depending on their geographical origin (*p* < 0.05). The mean L* values ranged from 13.36 ± 0.07 to 14.85 ± 0.02, with the highest value observed in the Ghana‐origin liquor and the lowest in the Côte d'Ivoire‐origin liquor. Statistical differences (*p* < 0.05) were observed among the samples in terms of L* values, indicating that brightness was influenced by the origin of the beans. As shown in Table 1, the mean hue angle (*h*°) values ranged from 47.29 ± 0.44 to 49.16 ± 0.83, with the highest value observed in the Côte d'Ivoire origin liquor and the lowest in the Ecuador origin liquor. The *h*° values differed between the Ecuador and Côte d'Ivoire samples (*p* < 0.05), whereas the Ghana sample showed comparable *h*° values to both the Ecuador and Côte d'Ivoire samples. The chroma (C*) values ranged from 22.23 ± 0.21 to 23.09 ± 0.48, with the highest values recorded for the Côte d'Ivoire samples and the lowest for the Ghana origin liquor. The Côte d'Ivoire liquor differed from both the Ecuador and Ghana liquors in terms of C* values (*p* < 0.05), whereas no difference was observed between the Ecuador and Ghana liquor. Since cocoa liquor is directly produced from cocoa beans, the color characteristics of the beans largely influence the color of the resulting liquor. The variations in color values among the samples might be attributed to differences in the geographical origin of the cocoa beans, which are linked to the variations in genetic profile and roasting behavior.

Cocoa butter content expressed on a dry matter basis varied substantially based on geographical origin (*p* < 0.05). Ghana cocoa liquor exhibited the highest value (53.81%), followed by Ecuador (53.14%) and Côte d'Ivoire (52.87%). Although the observed differences were relatively small, they were statistically significant, indicating that geographical origin contributed to variations in lipid content. These differences may be associated with genotype, environmental growing conditions, and post‐harvest practices, all of which have previously been reported to influence lipid accumulation in cocoa beans. Since all cocoa liquors were obtained from the same supplier and processed under standardized industrial conditions, the observed differences are likely attributable primarily to origin‐related factors rather than processing variability. The values are lower than those found by de Souza Silveira et al. (2025), who analyzed the fat content of two varieties of cocoa liquors: Parazinho and PS1319 at different harvesting times. They reported that the cocoa butter content of the cocoa liquors changed between 43.57% and 50.58% (g/g) depending on the harvesting time and variety of the cocoa beans.

### 3.2 Properties of Cocoa Butters

#### 3.2.1 Solid Fat Content

**FIGURE 2:**
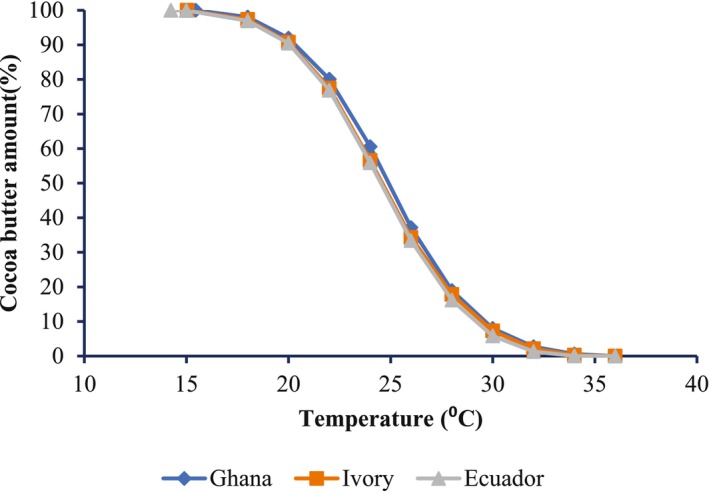
Percent solid fat versus temperature chart of cocoa butters from different origins.

Figure 2 shows the relationship between temperature and solid fat content in cocoa butters derived from cocoa liquors of three different geographical origins. Solid fat content is the percentage ratio of solid and liquid phases in a fat sample at varying temperatures and is a key factor affecting texture, physical stability, and sensory characteristics (Ribeiro et al. 2009). SFC level below 25°C indicates the fat's hardness, whereas SFC values between 25°C and 35°C indicate its resistance to heating. At temperatures of 27°C–33°C, the fat melts, releasing the product's flavor and freshness in the mouth. A high SFC above 35°C may suggest the existence of a solid fat fraction, leading to an unpleasant waxy residue upon tasting (Torbica et al. 2006). Quality chocolate should melt completely at body temperature and leave no solid residue in the mouth. The melting profile of cocoa butter, a key component of chocolate, directly affects the texture and melting behavior of the final product.

The onset temperatures of the samples were determined as 14.24°C for Ecuadorian cocoa butter, 15.46°C for Ghanaian cocoa butter, and 15.04°C for Ivorian cocoa butter. The results show that cocoa butter obtained from Ecuadorian cocoa liquor begins to melt at a slightly lower temperature compared to that obtained from Côte d'Ivoire and Ghana.

These differences in the beginning of the melting point may be related to the geographical origin of the cocoa bean; this can affect the fatty acid composition, triacylglycerol profile, and crystal polymorphisms of cocoa butter. The difference in solid fat content (ΔS) between 25°C and 35°C is an important and practical industrial parameter for evaluating product quality. At temperatures above 35°C, SFC creates a waxy texture on the palate that is easily detectable. In order for cocoa butter to be used in the production of chocolate in tropical climates, it must be hard and brittle at room temperature (SFC exceeding 50% at 25°C), have appropriate melting properties in the mouth, and do not contain waxy residue (Ribeiro et al. 2012). Ribeiro et al. (2012) reported that Brazilian cocoa butter exhibits SFC values of 80%–90% at 10°C and 0% at 35°C, demonstrating a melting behavior typical of well‐processed cocoa butter. In this study, it was observed that all cocoa butter melted completely at 35°C–36°C, which is in line with the melting profiles of high‐quality cocoa butters used in chocolate production.

#### 3.2.2 Fatty Acid Composition

The statistical analysis of the fatty acid composition of cocoa oils obtained from cocoa liquors of different geographical origins is presented in Table 2. In all samples, the dominant fatty acids are C18:0 (stearic acid), C18:1 (oleic acid), and C16:0 (palmitic acid), which together constitute more than 90% of the total fatty acids. Polyunsaturated fatty acids, particularly C18:2 (linoleic acid) and C18:3 (α‐linolenic acid) were found at relatively low levels, while the remaining fatty acids were detected in trace amounts or very low quantities.

**TABLE 2:** Fatty acid composition of cocoa butters from different geographical origin.

Fatty acid type	Ecuador	Ghana	Côte d'Ivoire
C4:0 (butanoic acid)	0.01 ± 0.01^a^	0.05 ± 0.01^b^	0.03 ± 0.01^a^
C6:0 (caproic acid)	0.02 ± 0.01^a^	0.04 ± 0.01^a^	0.03 ± 0.01^a^
C8:0 (caprylic acid)	0.02 ± 0.01^a^	0.04 ± 0.01^a^	0.02 ± 0.01^a^
C14:0 (myristic acid)	0.12 ± 0.02^a^	0.36 ± 0.01^b^	0.23 ± 0.02^c^
C14:1 (myristoleic acid)	0.05 ± 0.02^a^	0.03 ± 0.01^a^	0.05 ± 0.01^a^
C16:0 (palmitic acid)	26.70 ± 0.50^a^	28.66 ± 0.35^b^	26.16 ± 0.30^a^
C16:1 (palmitoleic acid)	0.27 ± 0.04^a^	0.28 ± 0.04^a^	0.26 ± 0.02^a^
C17:0 (margaric acid)	0.24 ± 0.02^a^	0.23 ± 0.02^a^	0.24 ± 0.02^a^
C17:1 (heptadecanoic acid)	0.03 ± 0.02^a^	0.03 ± 0.02^a^	0.04 ± 0.02^a^
C18:0 (stearic acid)	36.75 ± 0.40^a^	34.92 ± 0.47^b^	37.17 ± 0.11^a^
C18:1 (oleic acid)	32.58 ± 0.11^a^	32.47 ± 0.31^a^	32.36 ± 0.28^a^
C18:2 (linoleic acid)	2.78 ± 0.10^a^	2.87 ± 0.06^a^	2.79 ± 0.12^a^
C18:3 n‐3 (α‐linolenic acid)	0.06 ± 0.02^a^	0.06 ± 0.02^a^	0.06 ± 0.02^a^
C20:0 n‐6 (arachidicacid)	1.12 ± 0.10^a^	1.07 ± 0.08^a^	1.16 ± 0.06^a^
C24:0 (lignoceric acid)	0.14 ± 0.04^a^	0.09 ± 0.02^ab^	0.07 ± 0.01^b^

*Note:* Values followed by different letters are significantly different (*p <* 0.05) (Tukey multiple range test). Values are mean ± standard deviation The cocoa liquors represent commercially supplied bulk materials processed under standardized chocolate manufacturing conditions.

The C18:0 (stearic acid) content was found to vary between 34.92% ± 0.47% and 37.17% ± 0.11%, with the highest value observed in Ivory Coast cocoa butter and the lowest value in the Ghana sample. While there was no significant difference between the Ecuador and Ivory Coast samples (*p* > 0.05), a significant difference was determined between the Ecuador–Ghana and Ghana–Ivory Coast samples (*p* < 0.05). The C18:1 (oleic acid) content varied between 32.36% ± 0.28% and 32.58% ± 0.11%, with the highest mean value recorded in the Ecuador–origin cocoa butter and the lowest in the Côte d'Ivoire–origin sample. Statistical analysis indicated no significant differences among the three cocoa butter samples (*p* > 0.05). For C16:0 (palmitic acid), values ranged from 26.16% ± 0.30% to 28.66% ± 0.35%, with the highest level found in the Ghana–origin cocoa butter and the lowest in the Côte d'Ivoire–origin sample. While no significant difference was observed between the Ecuador and Côte d'Ivoire samples (*p* > 0.05), significant differences were detected between the Ecuador–Ghana and Ghana–Côte d'Ivoire samples (*p* < 0.05). The C18:2 (linoleic acid) content ranged from 2.78% ± 0.10% to 2.87% ± 0.06%, with the highest value observed in the Ghana–origin cocoa butter and the lowest in the Ecuador–origin sample. The C18:2 content was comparable among the three geographical origins (*p* > 0.05).

Similarly, the C20:0 (arachidic acid) content ranged from 1.07% ± 0.08% to 1.16% ± 0.06%. Although slight variations were observed between the samples, these differences were statistically insignificant (*p* > 0.05).

The SFC trends illustrated in Figure 2 are in agreement with the fatty acid profiles reported in Table 2, suggesting that the observed differences in solid fat content may be associated with variations in fatty acid composition among the cocoa butter samples (Ornla‐ied et al. 2022). de Souza Silveira et al. (2025) reported a fatty acid profile comparable to that of cocoa butter, consistent with the findings of the present study. In both the literature and this work, palmitic, stearic, and oleic acids were identified as the predominant fatty acids, reflecting the characteristic composition of cocoa butter and supporting its classification as a high‐quality cocoa fat (Balcázar‐Zumaeta et al. 2026).

#### 3.2.3 Acidity and Peroxide Value

Cocoa butter is stable fat due to its highly saturated fatty acid content. However, oxidation may occur during roasting, resulting in changes in free fatty acid content, peroxide value, volatile organic compound composition, etc. (Peña‐Correa et al. 2024). The free fatty acidity and peroxide value of cocoa butters extracted from cocoa liquors of different geographical origins are presented in Table 3. The highest FFA value was observed in cocoa butter obtained from Ecuador‐origin cocoa liquor with 2.60% ± 0.07% oleic acid. In contrast, the lowest value was recorded for the Ghana‐origin sample with 1.62% ± 0.04% oleic acid. The Codex Alimentarius Standard for Cocoa Butter (CXS 86‐1981) defines that cocoa butter must have a free fatty acid content (as oleic acid) lower than 1.75% (g/g). Only cocoa butter from Ghana was found to comply with the Codex limit.

**TABLE 3:** Average values of free fatty acidity (FFA) and peroxide value (PV).

Origin	Free fatty acidity (oleic acid %) (w/v)	Peroxide value (meq/kg oil)
Ecuador	2.60 ± 0.07^a^	4.78 ± 0.23^a^
Ghana	1.62 ± 0.04^b^	3.91 ± 0.04^b^
Côte d'Ivoire	2.14 ± 0.10^c^	6.92 ± 0.05^c^

*Note:* Values followed by different letters are significantly different (*p <* 0.05) (Tukey multiple range test). Values are mean ± standard deviation. The cocoa liquors represent commercially supplied bulk materials processed under standardized chocolate manufacturing conditions.

Elevated FFA levels are generally associated with enzymatic or microbial lipolysis occurring during post‐harvest operations such as fermentation, drying, and storage (Peña‐Correa et al. 2024). Accordingly, the observed differences in FFA values among cocoa butters extracted from cocoa liquors of different geographical origins may be attributed to variations in post‐harvest handling practices, fermentation intensity, storage duration, and climatic conditions influencing bean composition and enzymatic activity. Also, triacylglycerols in cocoa beans are primarily hydrolyzed into free fatty acids, diacylglycerols, and monoacylglycerols by enzymatic processes during fermentation and post‐harvest (Afoakwa et al. 2014).

Foubert et al. (2004) investigated the influence of chemical composition on the isothermal crystallization behavior of cocoa butter. They reported FFA contents (expressed as % oleic acid) ranging from 1.16% to 2.77% among 20 cocoa butters of different origins. Specifically, FFA values of 2.18% and 1.91% were reported for Côte d'Ivoire cocoa butters obtained from two suppliers, while the Ecuador‐origin sample exhibited an FFA content of 1.18%. These values are in good agreement with the range obtained in the present study, supporting that the FFA contents of the cocoa butters analyzed fall within typical limits for well‐processed cocoa products.

The peroxide values (PV) of cocoa oils ranged from 3.91 ± 0.04 to 6.92 ± 0.05 meq O_2_/kg fat; the highest value was found in the Ivory Coast sample and the lowest value was found in the Ghana sample (Table 3). The peroxide value indicates the degree of primary lipid oxidation and the formation of peroxides and hydroperoxides during oxidative degradation. The relatively low PV values of cocoa oils suggest that they have not undergone excessive oxidation and have been stored under appropriate conditions. However, differences between samples may be related to geographical origin and/or post‐harvest processing which affect oxidative stability through changes in unsaturated fatty acid composition and natural antioxidants. Zyzelewicz et al. (2014) examined the effect of roasting conditions on oxidative changes in cocoa butter extracted from Forastero cocoa beans cultivated in Togo and reported PVs ranging from 2.5 to 20 meq O_2_/kg fat depending on air velocity, temperature, and humidity during roasting. The PV values obtained in the present study fall well within this range, indicating that the cocoa butters analyzed exhibited good oxidative quality and were comparable to values reported in previous studies. Overall, variability in origin, bean health, fermentation time, moisture/humidity during storage, and roasting/pressing conditions of cocoa liquors results in significantly varied free fatty acid and peroxide value in the cocoa butters extracted from them (Zyzelewicz et al. 2013; Mounjouenpou et al. 2018; Sari et al. 2025).

### 3.3 Properties of Chocolate From Different Cocoa Liquors

#### 3.3.1 Color

In dark chocolates, L* values ranged from 13.97 ± 0.05 to 16.32 ± 0.05, and statistical differences were observed among the technical measurements obtained from the single production batch used in this preliminary study (Table 4). The Ghana‐origin dark chocolate showed a higher L* value, indicating a lighter appearance. In milk chocolates, L* values were higher than those of dark chocolates and ranged from 38.61 ± 0.08 to 40.28 ± 0.09. These results suggest that differences in the lightness of milk chocolates may be associated with the lightness of the cocoa liquor. Chroma (C*) values showed limited variation among dark chocolate samples. In milk chocolates, C* values were similar among the samples. Hue angle (*h*°) values were also similar within each chocolate type, indicating that the color tone was preserved. Overall, milk chocolates showed higher L* and C* values than dark chocolates, due to the ingredients used in the formulation, such as milk solids and the fat phase. These results indicate that color differences is more evident in dark chocolates from different cocoa origins, whereas milk chocolates had more uniform color characteristics. Previous studies reported that higher phenolic acid content and melanoidin formation during roasting led to darker coloration due to non‐enzymatic browning reactions (Afoakwa et al. 2007). Differences observed among cocoa origins in the present study may also be associated with compositional variations previously reported in origin‐based cocoa characterization studies (Araujo et al. 2014), although detailed chemical profiling was beyond the scope of this work.

**TABLE 4:** Color parameters of the produced chocolate samples.

Type	Origin	L*	a*	b*	C*	*h°*
Dark chocolate	Ecuador	13.97 ± 0.05^c^	10.89 ± 0.03^a^	13.27 ± 0.01^c^	17.17 ± 0.51^b^	50.61 ± 1.36^a^
	Ghana	16.32 ± 0.05^a^	10.81 ± 0.01^a^	14.17 ± 0.02^b^	17.82 ± 0.35^ab^	52.64 ± 0.74^a^
	Côte d'Ivoire	15.02 ± 0.11^b^	11.22 ± 0.05^b^	15.06 ± 0.02^a^	18.78 ± 0.29^a^	53.31 ± 1.35^a^
Milk chocolate	Ecuador	40.28 ± 0.09^a^	11.90 ± 0.01^c^	20.38 ± 0.06^b^	23.60 ± 0.09^c^	59.71 ± 0.48^b^
	Ghana	38.61 ± 0.08^b^	12.76 ± 0.01^c^	20.38 ± 0.01^b^	27.05 ± 0.21^a^	61.85 ± 0.31^a^
	Côte d'Ivoire	38.65 ± 0.10^b^	12.29 ± 0.03^c^	22.81 ± 0.05^a^	25.91 ± 0.13^b^	61.69 ± 0.15^a^

*Note:* Values followed by different letters are significantly different (*p <* 0.05) (Tukey multiple range test). Values are mean ± standard deviation. Statistical comparisons were performed using technical replicates from a single production batch for each formulation; therefore, the statistical inference is limited to this experimental batch.

#### 3.3.2 Texture Properties

Texture properties are critical mechanical characteristics of chocolate, since they are closely associated with the product's sensory evaluation and provide a basis for predicting its rheological behavior during processing (Ostrowska‐Ligeza et al. 2019). Consumers mainly assess chocolate texture during breaking and chewing it, considering characteristics such as hardness, strength, melting behavior, and adhesiveness. Additionally, they assess viscosity, smoothness, fineness, and the sensation of dispersed solid particles while holding fragmented and melted chocolate between the tongue and palate. Thus, the measurement of textural properties is important for evaluating chocolate quality and optimizing process control in the chocolate process (Balcázar‐Zumaeta et al. 2026). The texture of chocolate depends on the type and proportions of raw materials used in its formulation. The cocoa butter added to the formulation and its substitutes play a particularly critical role (Zhao et al. 2018).

The textural properties of dark and milk chocolates produced from cocoa liquors of different geographical origins are given in Table 5. The bending strength values of the dark chocolate samples ranged from 23.01 ± 2.10 to 24.33 ± 3.82 N, showing only limited variation among the samples. Similarly, the bending strength values of the milk chocolate samples ranged from 21.94 ± 3.24 to 23.23 ± 3.23 N, with comparable values observed among the samples. These results indicate that the flexural strength is similar regardless of the cocoa origin. This similarity suggests that the flexural behavior is determined by structural factors such as total fat content and tempering conditions, rather than the origin of the cocoa liquor.

**TABLE 5:** The textural properties of dark and milk chocolates produced.

Origin	Dark chocolate		Milk chocolate	
	Bending force (N)	Hardness (N)	Bending force (N)	Hardness (N)
Ecuador	24.33 ± 3.82^a^	23.49 ± 1.28^a^	21.94 ± 3.24^a^	21.86 ± 3.08^a^
Ghana	24.25 ± 6.23^a^	27.59 ± 1.84^b^	22.97 ± 6.14^a^	20.24 ± 1.79^a^
Côte d'Ivoire	23.01 ± 2.10^a^	23.91 ± 1.08^a^	23.23 ± 3.23^a^	22.33 ± 7.03^a^

*Note:* Values followed by different letters are significantly different (*p <* 0.05) (Tukey multiple range test). Values are mean ± standard deviation. Statistical comparisons were performed using technical replicates from a single production batch for each formulation; therefore, the statistical inference is limited to this experimental batch.

In terms of penetration hardness, the Ghana dark chocolate exhibited the highest hardness value (27.59 ± 1.84 N) among the dark chocolate samples. The penetration hardness values of the milk chocolate samples ranged from 20.24 ± 1.79 to 22.33 ± 7.03 N, showing only limited variation among the samples. The comparable bending strength and penetration hardness values observed in the milk chocolate samples suggest a relatively homogeneous texture profile within this preliminary study. This may be attributed to the presence of milk powder, which has been reported to stabilize the crystal structure and reduce variations associated with the cocoa liquor. The dark chocolate samples generally exhibited higher penetration hardness values than the milk chocolate samples. Similar observations have been reported by Ostrowska‐Ligeza et al. (2019).

#### 3.3.3 Rheological Properties of Chocolates

The rheological properties of chocolate are directly related to its viscosity, consistency, and mouthfeel; they directly affect both product quality and shelf life. Furthermore, rheological behavior influences how chocolate behaves in the mouth and its perception during consumption; this includes possible differences in odor release (Toker et al. 2023). The rheological properties of chocolate are primarily influenced by particle size distribution and formulation composition. These factors affect the final texture and melting profile of the product and have a critical role in food processes (Gonçalves and Lannes 2010).

**FIGURE 3:**
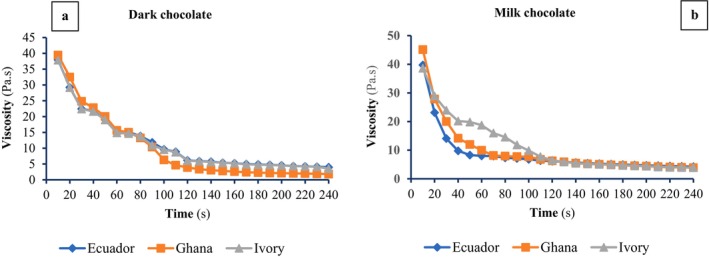
Viscosity‐time graph for chocolates produced using cocoa liquor from three different origins. (a) Dark chocolate; (b) milk chocolate.

**FIGURE 4:**
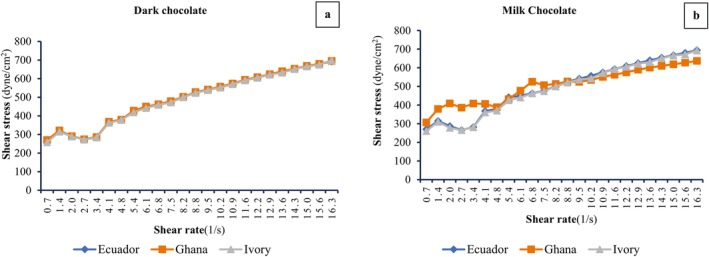
Shear stress‐shear ratio graph for chocolates produced using cocoa liquor from three different origins. (a) Dark chocolate, (b) milk chocolate.

The viscosity–time and shear stress–shear rate curves of milk and dark chocolates produced using cocoa liquor from three different geographical origins are presented in Figures 3 and 4, respectively. As shown in Figure 3a,b, the viscosity of both chocolate types produced from different cocoa liquor origins decreased over time. Similarly, Zarić et al. (2024) showed that in all chocolate types (dark, white, milk, ruby), viscosity decreases with increasing shearing rate. The viscosity values of the dark chocolate samples ranged from 37.46 ± 0.48 to 38.52 ± 0.86 Pa s. The highest viscosity value was observed for dark chocolate prepared with Ghanaian cocoa liquor (38.52 ± 0.86 Pa s), while the lowest was recorded for the sample prepared with Ecuadorian cocoa liquor (37.46 ± 0.48 Pa s).

The viscosity values for milk chocolate samples varied between 38.48 ± 0.19 and 40.22 ± 4.28 Pa s. The highest viscosity value was observed for the milk chocolate prepared with Ghanaian cocoa liquor (40.22 ± 4.28 Pa s), whereas the lowest was recorded for the sample prepared with Côte d'Ivoire cocoa liquor (38.48 ± 0.19 Pa s). Overall, the viscosity values were comparable among the chocolate samples prepared with cocoa liquors from Ecuador, Ghana, and Côte d'Ivoire. Glicerina et al. (2016) compared the microstructure and rheological properties of white, milk, and dark chocolates and reported that white chocolates showed the lowest viscosity values, while milk and dark chocolates exhibited higher viscosity. Additionally, it has been reported that milk chocolates have lower viscosity values compared to dark chocolates. Contrary to these findings, the current study showed that milk chocolates exhibit higher viscosity values than their dark chocolate counterparts, regardless of the cocoa source. Siow et al. (2017) investigated the physical, rheological, and sensory properties of dark chocolates produced with cocoa butter substitutes; they determined that the apparent viscosity decreased with increasing shear rate, while the yield stress increased.

The current study revealed that chocolate samples exhibit pronounced shear thinning behavior. In both dark and milk chocolate samples produced from cocoa liquors of various geographical origins, shear stress increased nonlinearly with increasing shear rate, as seen in Figure 4a,b. This situation indicates that under the applied shear force, structural degradation and particle alignment occur in the continuous oil phase. Moisture content, although not measured, is assumed to be low and comparable across samples, and therefore its influence on rheological differences is considered limited under identical processing conditions.

### 3.4 Chemometric Analysis of Cacao Liquors and Chocolates

**FIGURE 5:**
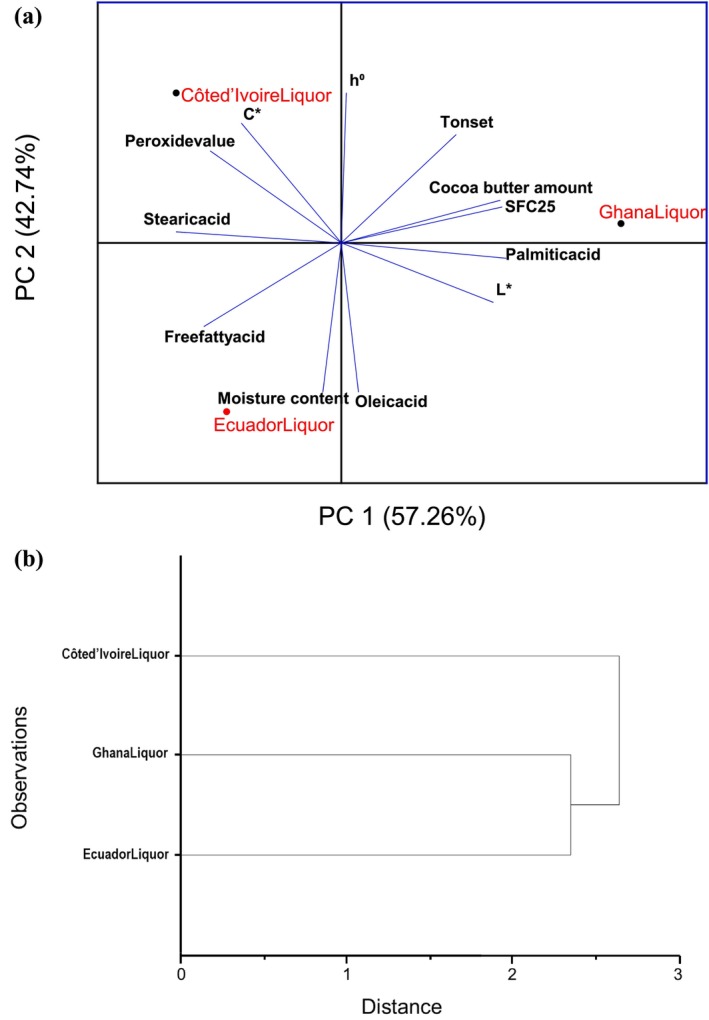
Principal component analysis biplot (a) and hierarchical cluster analysis (b) of cocoa liquor samples from different geographical origins based on physicochemical, thermal, and fatty acid composition parameters.

The principal component analysis (PCA) revealed a clear discrimination among cocoa liquor samples originating from Ecuador, Ghana, and Côte d'Ivoire, with the first two principal components explaining 100% of the total variance (PC1 = 57.26% and PC2 = 42.74%) (Figure 5a). The distribution of samples across the PCA plot indicated that geographical origin exerted a substantial influence on the physicochemical, thermal, and lipid composition characteristics of cocoa liquors. Ghana liquor was positioned on the positive side of PC1 and showed a strong association with cocoa butter content, solid fat content at 25°C (SFC25), palmitic acid concentration, and Tonset values. This clustering suggests that Ghanaian cocoa liquor possesses a lipid profile favoring higher solid fat fractions and enhanced thermal stability. The association between cocoa butter content and SFC25 further indicates a greater proportion of crystallizable lipid fractions, which may contribute positively to processing performance and texture development in subsequent chocolate manufacture.

In contrast, Côte d'Ivoire liquor was characterized by positive PC2 values and was closely associated with peroxide value, stearic acid, chroma (C*), and hue angle (*h*°). The relationship with peroxide value suggests that oxidative characteristics contributed significantly to the differentiation of this sample. Simultaneously, the association with color parameters indicates that pigment composition and post‐harvest processing conditions may have influenced its unique position within the PCA plot. Ecuador liquor was separated in the negative region of both PC1 and PC2 and was strongly related to moisture content, free fatty acids, and oleic acid. The proximity of free fatty acids and moisture content vectors suggests that hydrolytic reactions may have played an important role in shaping the compositional characteristics of this sample. Elevated moisture levels can accelerate lipolytic activity during post‐harvest handling and storage, potentially leading to increased free fatty acid formation.

The hierarchical cluster analysis (Figure 5b) supported the PCA findings by revealing a distinct separation of Côte d'Ivoire liquor from the other origins, while Ghana and Ecuador samples exhibited relatively greater similarity. This clustering pattern indicates that although all samples originated from major cocoa‐producing regions, differences in genotype, environmental conditions, fermentation practices, and drying procedures significantly affected the final quality attributes of the cocoa liquors.

**FIGURE 6:**
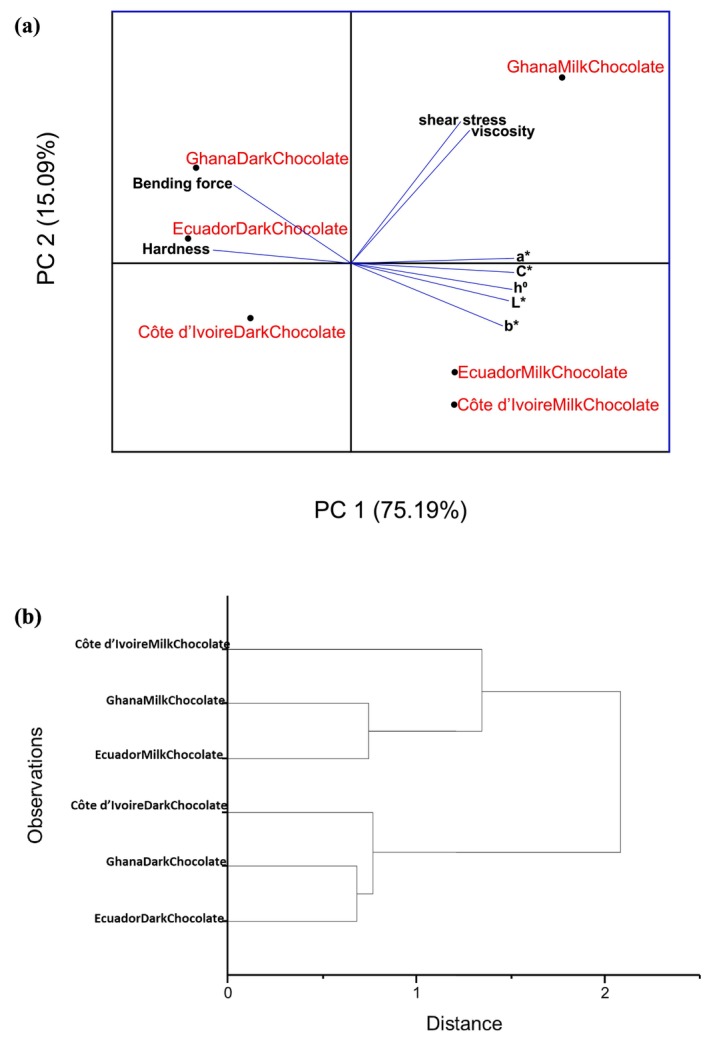
Principal component analysis (PCA) biplot (a) and hierarchical cluster analysis (b) of dark and milk chocolates produced from cocoa liquors of different geographical origins based on color, texture, and rheological properties.

For the chocolate samples, the PCA demonstrated that the first two principal components accounted for 90.28% of the total variability (PC1 = 75.19% and PC2 = 15.09%) (Figure 6a), indicating that the selected physicochemical, color, textural, and rheological parameters effectively described the differences among samples. The PCA plot revealed that PC1 was primarily influenced by color‐related attributes (L*, a*, b*, C*, and *h*°), whereas PC2 was mainly associated with rheological properties, particularly viscosity and shear stress. The dominance of color parameters along PC1 highlights the substantial contribution of formulation composition, especially the presence of milk solids, to the visual characteristics of chocolate products. Milk chocolate samples were generally located on the positive side of PC1, reflecting their close association with color parameters. Among them, Ghana milk chocolate exhibited a distinctive position in the upper‐right quadrant and was strongly correlated with viscosity and shear stress. This result suggests that the cocoa liquor originating from Ghana contributed to the formation of a chocolate matrix with enhanced flow resistance and structural organization, potentially due to differences in fat composition and solid fat content. Dark chocolate samples were predominantly positioned on the negative side of PC1 and were associated with hardness and bending force. The close proximity of these texture‐related variables indicates that mechanical resistance was a key characteristic distinguishing dark chocolates from milk chocolates. The higher hardness values observed in dark chocolates may be attributed to their greater cocoa solid content and lower proportion of milk‐derived components, which influence crystal network formation and structural rigidity (Zarić et al. 2024).

The hierarchical cluster analysis (Figure 6b) further confirmed the PCA results by separating the samples into two major groups. One cluster consisted predominantly of milk chocolates, whereas the second cluster contained dark chocolate samples. Within each group, samples showed additional sub‐clustering by geographical origin. These findings suggest that chocolate formulation exerted the primary influence on sample discrimination, while the geographical origin of cocoa liquor contributed as a secondary but still significant source of variation. Overall, the combined PCA and HCA results demonstrate that both raw material origin and product formulation affect chocolate quality characteristics; however, formulation‐related factors appear to have a more pronounced impact on color, texture, and rheological behavior. Given the limited number of samples included in the multivariate analysis (*n* = 3 cocoa liquors and *n* = 6 chocolate samples), the PCA and HCA should be regarded as exploratory analyses. Therefore, the clustering patterns observed should be interpreted as descriptive rather than confirmatory. In addition, the 100% cumulative variance explained by PC1 and PC2 for the cocoa liquor dataset (Figure 5a) is likely influenced by the small sample size.

## 4 Conclusion

In this study, cocoa liquors and cocoa butter originating from different geographical regions, which are widely used in the confectionery industry, were examined in terms of their chemical and physical properties. Additionally, their potential effects on the textural and rheological properties of milk and dark chocolate were investigated. It has been determined that cocoa liquors from different geographical origins and the cocoa butter obtained from them exhibit physicochemical differences in terms of oxidative stability, free fatty acids, and color. According to the results of this study, the chocolate composition is also effective on the final product properties, in addition to the inherent differences in cocoa products from different origins. The findings show that the addition of milk components and extra fat phases in milk chocolate systems substantially reduces variances caused by origin, resulting in highly uniform textural, colorimetric, and rheological characteristics. Conversely, the matrix of dark chocolate is more susceptible to these source variances, mostly manifested as changes in structural hardness and surface luminosity. Ultimately, irrespective of cocoa origin, the mechanical and flow characteristics of chocolates are predominantly affected by industrial processing and formulation rather than cocoa origin. The milk and dark chocolates were distinctly separated, with dark chocolates exhibiting higher hardness and bending force, whereas milk chocolates were associated with color and rheological characteristics. Among milk chocolates, the Ghana sample exhibited elevated viscosity and shear stress, whereas the Ecuador and Côte d'Ivoire samples were more closely associated with color characteristics. A limitation of this study is that it was based on a single production batch. Further studies, incorporating independent process replicates, together with aroma profiling and sensory evaluation, are needed to provide a more comprehensive understanding of the effect of cocoa liquor origin on chocolate quality.

## Author Contributions

**Onur Ozdemir:** conceptualization, writing – original draft, formal analysis. **Hasene Keskin‐Cavdar:** conceptualization, writing – original draft, data curation, formal analysis, methodology, writing – review and editing. **Mustafa Umit Unal:** conceptualization, investigation, funding acquisition, supervision, methodology, project administration, writing – review and editing. **Suleyman Polat:** conceptualization, writing – review and editing, visualization, methodology, formal analysis, writing – original draft, supervision.

## Funding

This study was financially supported by the Çukurova University Scientific Research Projects Unit (BAP) under Project No. FYL‐2018‐11330. The Scientific and Technological Research Council of Türkiye supported publishing this manuscript as open access.

## Conflicts of Interest

One of the authors was employed by Sölen Chocolate during the research and pilot‐scale production phase of the study. However, the companies had no role in the study design, data collection and analysis, interpretation of data, decision to publish, or preparation of the manuscript. The authors declare that they have no competing financial interests or personal relationships that could have appeared to influence the work reported in this paper.

## Data Availability

The data supporting the findings of this study are available within the article.
